# Shrinking multiplexed orbital angular momentum to the nanoscale

**DOI:** 10.1038/s41377-021-00668-6

**Published:** 2021-10-28

**Authors:** Chao He, Yijie Shen, Andrew Forbes, Martin J. Booth

**Affiliations:** 1 Department of Engineering Science, University of Oxford, Parks Road, Oxford, OX1 3PJ UK; 2 Optoelectronics Research Centre, University of Southampton, Southampton, SO17 1BJ UK; 3 School of Physics, University of the Witwatersrand, Private Bag 3, Johannesburg, 2050 South Africa

**Keywords:** Optics and photonics, Optical techniques

## Abstract

Orbital angular momentum interactions at the nanoscale have remained elusive because the phase structure becomes unresolved. Now researchers have shown how to overcome this with tightly focused beams, demonstrating a record-high six-dimensional encoding in an ultra-dense nanoscale volume.

The demand for ultrafast, condensed, broadband and secure transfer of energy and information is advancing at an unprecedented pace, with 6 G telecommunication already on its way. An enabler of this is to exploit light’s many degrees of freedom (DoF) for optical multiplexing technology, now an essential tool for modern information delivery. The use of ultrafast pulsed lasers has facilitated the transition of data recording from a surface layer to a 3D volume^1^. Advances in nanotechnology, especially plasmonic nanomaterials, have enabled light multiplexing to be performed with five DoFs^2^. An exciting prospect to overcome the impending data crunch^3^ is to use the spatial mode of light as an additional DoF, with orbital angular momentum (OAM) a popular candidate^4^. But modern optical information technology systems require miniaturised and integrated solutions, and here OAM has a problem: as one zooms into the nanoscale, the telltale helical structure of OAM gets lost as the light wave appears locally flat. Just as ants cannot easily tell whether they are walking along a straight rod or a helical corkscrew, nanostructured matter cannot discern the OAM of a spatial mode. In a recently published paper^5^, Xu Ouyang and co-authors overcome this challenge in an ingenious manner. They note that while the OAM phase itself cannot be detected, its influence on the resulting sub-wavelength polarisation structure can. In a tightly focussed OAM beam, fine polarisation ellipses arise with orientations dictated by the OAM charge. This orientation can be detected by nanostructures with a similar orientation. Using this as a tool, the authors demonstrate that six-dimensional optical OAM multiplexing can be realised at the nanoscale, exploiting wavelength, polarisation, and three spatial dimensions simultaneously (Fig. 1).

**Fig. 1:**
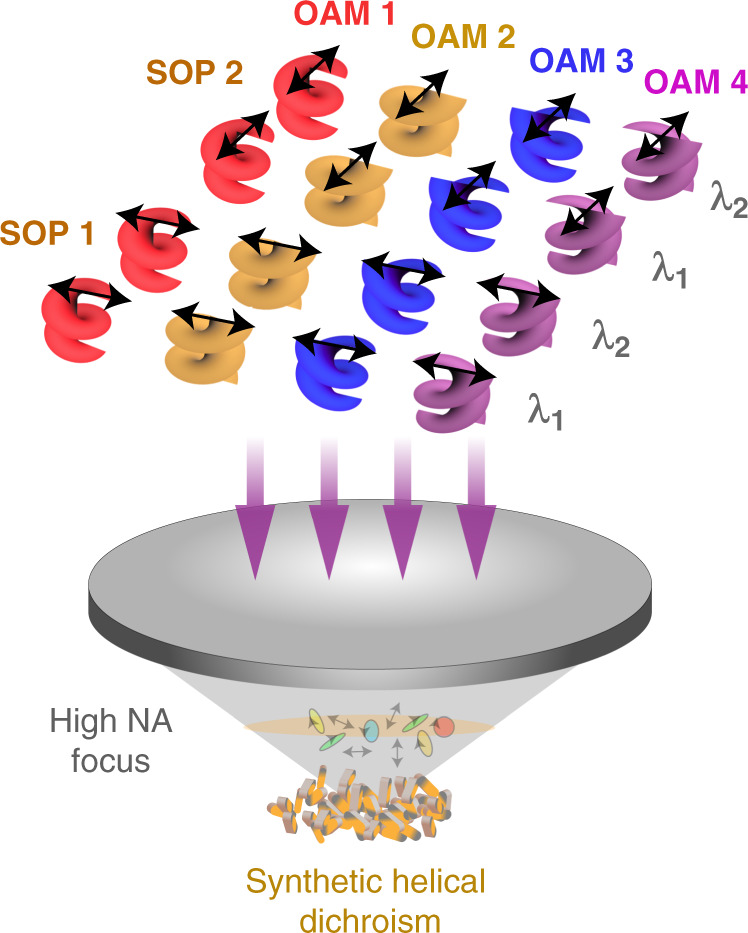
Illustrations of six-dimensional OAM multiplexing. SOP state of polarisation, λ wavelength

A combination of tight focusing and synthetic helical dichroism (HD) play important roles in this breakthrough. High numerical-aperture (NA) lenses, which are essential for tight beam focusing, feature intrinsic polarisation aberrations^6,7^ (for example, owing to Fresnel and geometric effects). One aspect of its overall representation is that in the nonparaxial vectorial electric field, spatial variations exist in the state of polarisation, which varies for different incident OAM beams. While commonly seen as a nuisance (e.g., for polarisation imaging) or overlooked (e.g., in optical multiplexing), this property is in contrast harnessed by the team in conjunction with the synthetic HD. Dichroism, a material property that induces diattenuation during light propagation, has been used in many research fields, ranging from quantum physics to clinical applications. In principle, it is associated with a difference in absorption coefficients for orthogonal polarisation states, resulting in different polarisation responses that are encoded in the corresponding materials. The team describes and leverages the synthetic HD of a nanostructure under the excitation of OAM beams with respect to the topological charge, linear polarisation, and wavelength. Such synthetic HD is polarisation-sensitive, hence can be utilised together with the different polarisation ellipses in the tightly focused beam that link with different OAM charges, enabling sensitive OAM multiplexing at the nanoscale. Compared with conventional HD in chiral structures, this synthetic HD is not restricted to opposite topological charges, hence provides more versatile possibilities^5,8^.

The team utilise these properties to demonstrate six-dimensional optical encryption with nanometric QR codes via OAM division regarding two wavelengths and orthogonal incident polarisation states. Their success also build upon plasmonic coupling effects that introduce hotspots with remarkably augmented local fields to enhance synthetic HD, and two-photon luminescence for an intuitive indication of the synthetic HD. These phenomena are of significant benefit in the encoding and decoding processes. Furthermore, the six-dimensional encoded images are retrieved from different layers of the coupled disordered nanoparticle aggregates. It is worth noting that the sensitivity of the photothermal deformation of gold nanorods and the two-photon luminescence contrast can both be boosted considerably by the enhanced HD, hence benefiting the multiplexing process via improved signals.

The results obtained by the team open exciting possibilities of multiplexing the OAM division for high-information capacity and security. Building upon these advances in six-dimensional multiplexing, there is still work to be done. The polarisation effects vary between different high NA lenses and these effects can disorder the traceable polarisation ellipses in the focal region. Hence, cross-talk could occur between different OAM channels. However, this is likely to be conquered with engineering solutions through lens optimisation. There are also prospects for further extensions, such as towards higher-dimensional structured light multiplexing. Recent advances in structured light^9^ have pushed the limits of what is possible. The present work highlights the exciting prospects in marrying structured light with structured matter, for control at scales from the large to the small.
